# Effective treatment of HIV-associated Kaposi sarcoma in the setting of immune-reconstitution inflammatory syndrome using intralesional bleomycin

**DOI:** 10.1016/j.jdcr.2022.07.016

**Published:** 2022-07-19

**Authors:** Brittany Ehlert, Rachel Delost, Vinaya Soundararajan, Gregory Delost, Danny Barlev

**Affiliations:** a Ohio University Heritage College of Osteopathic Medicine, Cleveland, Ohio; b Department of Dermatology, Apex Dermatology, Mentor, Ohio; c Department of Dermatology, University Hospitals Cleveland Medical Center, Cleveland, Ohio; d Department of Dermatology, Apex Dermatology, Mayfield Heights, Ohio

**Keywords:** AIDS, HAART, HIV, intralesional bleomycin, Kaposi sarcoma, ART, antiretroviral therapy, IRIS, immune-reconstitution inflammatory syndrome, IL, intralesional, KS, Kaposi sarcoma

## Introduction

Kaposi sarcoma (KS) is a neoplasm of endothelial cells that is caused by human herpesvirus-8. There are 4 different classifications of KS, including classic (present in elderly men or Mediterranean individuals), endemic (seen in individuals from sub-Saharan Africa), iatrogenic (immunosuppression/organ transplant-related), and epidemic (HIV-associated). KS is characterized by violaceus or dark macules, plaques, or nodules on the skin with potential involvement of the mouth, gastrointestinal tract, lungs, or lymph nodes.

First-line treatment for HIV-associated KS includes antiretroviral therapy (ART).^1^ A potential complication of ART includes immune-reconstitution inflammatory syndrome (IRIS).^2^ IRIS is a hyperinflammatory response against opportunistic infections, like human herpesvirus-8, that is typically present in the first 6 months of treatment of HIV and AIDS.^2^^,^^3^ The diagnostic criteria for IRIS include a patient with HIV receiving ART with either a decrease in HIV-1 RNA level and/or an increase in CD4^+^ cells from baseline, as well as clinical symptoms consistent with an inflammatory process but not consistent with an expected course of a previously or newly diagnosed opportunistic infection or drug side effect or toxicity.^2^ IRIS can be classified as “unmasking” or “paradoxical” depending on whether it leads to a new HIV-associated condition (unmasking) or worsening of a condition (paradoxical).^3^ Proposed diagnostic criteria for KS-IRIS include 2 or more of the following: an abrupt increase in several KS lesions, the appearance or exacerbation of lung opacities or lymphedema, as well as an increase in CD4^+^ cell count ≥ 50 cells/mm,^4^ and a decrease of >1 log in viral load once started combination ART.^2^ In addition to ART, systemic therapy may be needed in the setting or prevention of IRIS or the case of extensive disease.^1^ Reported systemic therapies include liposomal daunorubicin, bleomycin, vincristine, paclitaxel, etoposide, and liposomal doxorubicin.^4^^,^^5^ Bleomycin can be used intravenous, intramuscular, or intralesional (IL) to treat KS, but there are few reports discussing the efficacy of IL bleomycin, especially for HIV-associated KS in the setting of IRIS.^6^, ^7^, ^8^

We report a case on IL bleomycin treatment success for HIV-associated KS in the setting of IRIS.

## Case report

A 30-year-old man with a history of HIV (diagnosed in 2016), syphilis, and stroke (in the setting of Moyamoya disease) was previously on ART with Complera (emtricitabine/rilpivirine/tenofovir) in 2016 but stopped his medications and did not reestablish care until roughly 2019. In April 2021, his CD4 count was 184, and his viral load was <20, and in May 2021, he was started on ART, Biktarvy (bictegravir/emtricitabine/tenofovir alafenamide).

**Fig 1:**
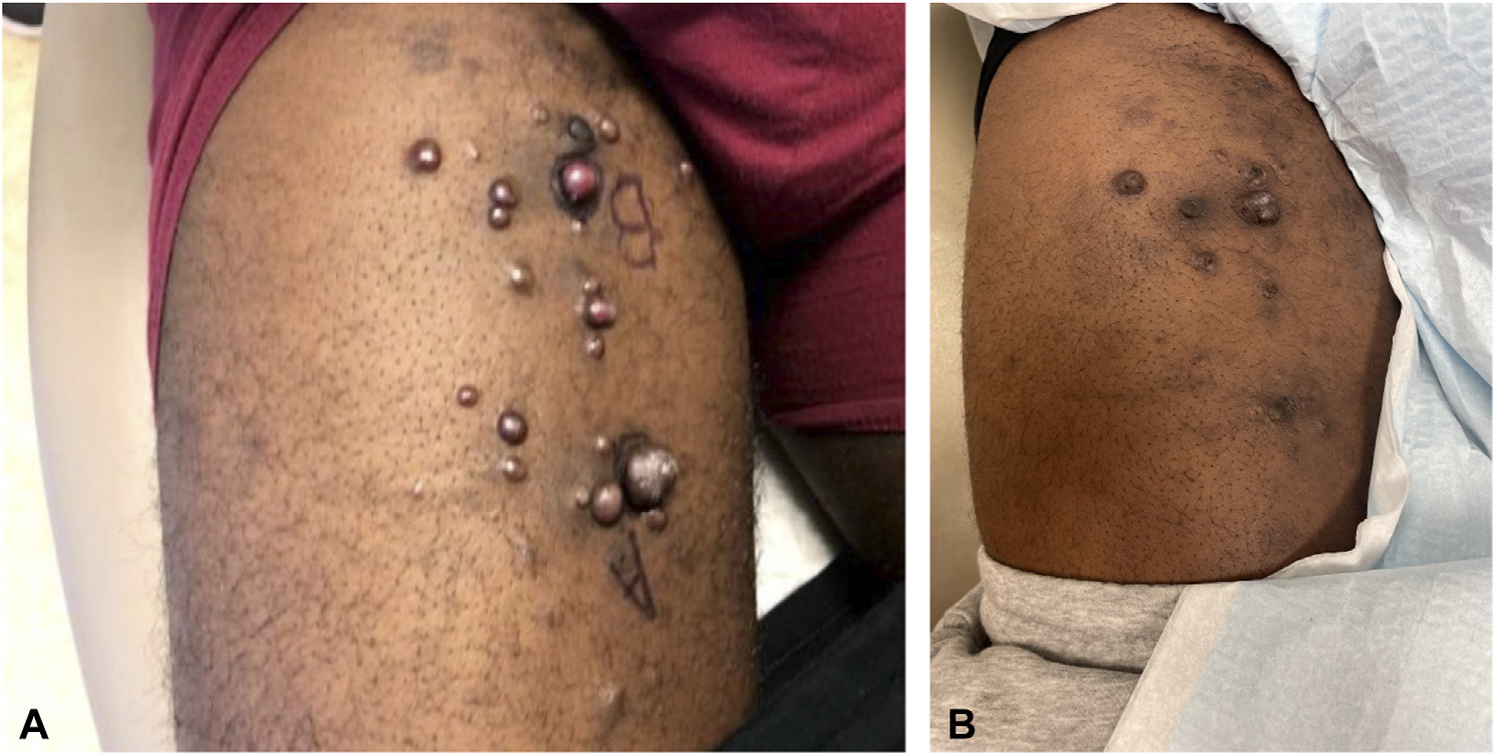
Kaposi sarcoma lesions on right forearm pre- and post-intralesional bleomycin (**A**) before treatment in October 2021 and (**B**) posttreatment in March 2022.

**Fig 2:**
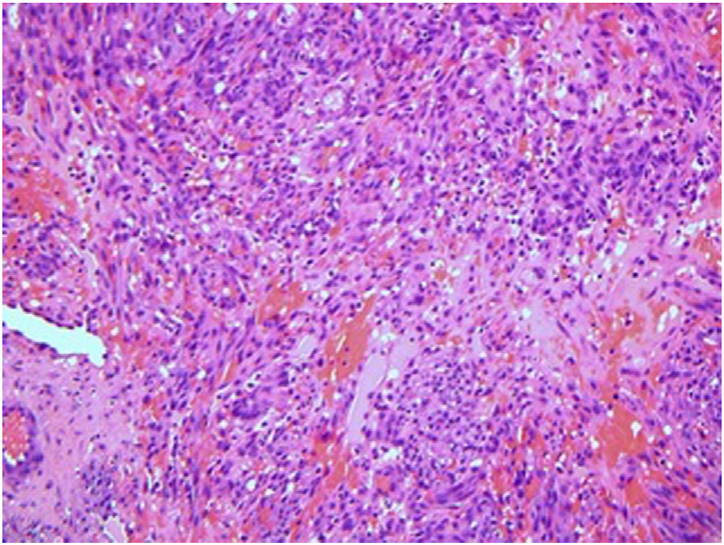
Hematoxylin and eosin stain demonstrate plump spindled cells admixed with blood vessels in the dermis. These cells are stained with antibodies against human herpesvirus 8. (Hematoxylin-eosin stain; original magnification: ×20).

In June 2021, he presented with 2 violaceous nodules on the right thigh (Fig 1, *A*), and a biopsy revealed KS (Fig 2). He also had leg swelling, in which computed tomography (CT) angiography for pulmonary embolism was done and showed enlarged bilateral axillary lymph nodes. At that time, the CD4 count was 251 and the viral load was <20.

Three months after starting ART, positron emission tomography/CT showed mild to moderately hypermetabolic bilateral axillary, external iliac chain, inguinal, and left popliteal fossa lymph nodes. Three weeks later, new skin lesions appeared on the right thigh (grouped pink to slightly violaceous papules to nodules, ranging in size from 5 mm-1.3 cm). It was recommended to start IL bleomycin in the setting of IRIS KS.

The patient received 4 total treatments of IL bleomycin in February and March of 2022. In his first treatment as a test dose, 0.5 cc of bleomycin sulfate 3 unit/mL was injected intradermally in 3 lesions (a total of 1.5 units). In the subsequent treatments, 3 weeks apart, there were approximately 10 lesions that required additional injections. Most were resolved after only 1 session and required ≤0.5 units. At the end of his treatment sessions, there were still 3 lesions that have not fully resolved, and it remains unclear if they reflect residual KS or scarring or fibrosis from bleomycin (Fig 1, *B*). These unresolved nodules appeared much smaller on the examination and will be monitored with continued follow-up with this patient. If the nodules increase in size or change in morphology, they will be biopsied.

## Discussion

The efficacy of IL bleomycin for HIV-associated KS has been reported in a few studies but not in the setting of KS-IRIS. One study in 1984 used 0.1 mg of IL bleomycin or IL vincristine and reported 80% complete regression with partial regression of the other 20% of 93 KS nodules, whereas another study in 1995 used IL bleomycin as monotherapy and found 87% of the 134 lesions showed complete or partial regression using a median dose of 1.5 mg per lesion.^7^^,^^9^ A case report in 2021 reported successful flattening of nodules on a patient’s foot after 6 months of IL bleomycin and cryotherapy that were previously unresponsive to other HIV-associated KS therapies with a continuing resolution of lesions after 1 year.^6^

Although our patient did not have evidence of lung opacities on chest CT, he did have lymphedema on examination. This combined with the abrupt onset of KS lesions after restarting ART is consistent with KS-associated IRIS. Monotherapy of IL bleomycin was used to treat the lesions and substantially decreased the size of the nodules. Because of the effectiveness of this treatment, cryotherapy was not used, although success was reported in a previous case. This knowledge is useful because of the potential side effect of significant posttreatment dyspigmentation seen with cryotherapy, especially in darker-skinned individuals.

This case supports prior research that IL bleomycin is an effective treatment for HIV-associated KS and provides further evidence that this treatment can also be used for HIV-associated KS in the setting of IRIS. Notably, it is possible that while IRIS caused paradoxical KS, immune reconstitution could have also been responsible for clearing the disease without the use of any therapeutic intervention. IL bleomycin should be considered in patients desiring treatment or for those who do not spontaneously improve from immune reconstitution.

## Conflicts of interest

None disclosed.
